# Single cell proteogenomic sequencing identifies a relapse‐fated AML subclone carrying *FLT3*‐ITD with CN‐LOH at chr13q

**DOI:** 10.1002/jha2.390

**Published:** 2022-02-24

**Authors:** TaeHyung Kim, Hyewon Lee, Jose‐Mario Capo‐Chichi, Myung Hee Chang, Young Seok Yoo, Gurbaksh Basi, Troy Ketela, Adam C. Smith, Anne Tierens, Zhaolei Zhang, Mark D. Minden, Dennis Dong Hwan Kim

**Affiliations:** ^1^ Division of Medical Oncology and Hematology Princess Margaret Cancer Centre Toronto Ontario Canada; ^2^ Department of Computer Science University of Toronto Toronto Ontario Canada; ^3^ The Donnelly Centre for Cellular and Biomolecular Research University of Toronto Toronto Ontario Canada; ^4^ Center for Hematologic Malignancies National Cancer Center Goyang Republic of Korea; ^5^ Department of Clinical Laboratory Genetics Genome Diagnostics Laboratory Medicine Program University of Toronto Toronto Ontario Canada; ^6^ Division of Oncology‐Hematology Department of Internal Medicine National Health Insurance Service Ilsan Hospital Goyang Republic of Korea; ^7^ Princess Margaret Genomics Centre Princess Margaret Cancer Centre Toronto Ontario Canada; ^8^ Laboratory Medicine Program University Health Network Toronto Ontario Canada; ^9^ Department of Laboratory Medicine and Pathobiology University of Toronto Toronto Ontario Canada; ^10^ Department of Molecular Genetics University of Toronto Toronto Ontario Canada; ^11^ Hans Messner Allogeneic Blood and Marrow Transplant Unit Princess Margaret Cancer Centre Toronto Ontario Canada

**Keywords:** AML, molecular diagnostics, prognostic factors, single cell

## Abstract

Internal tandem duplication of the Feline McDonough Sarcoma (FMS)‐like tyrosine kinase 3 (*FLT3*‐ITD) is one of the most clinically relevant mutations in acute myeloid leukemia (AML), with a high *FLT3*‐ITD allelic ratio (AR) (≥0.5) being strongly associated with poor prognosis. *FLT3*‐ITDs are heterogeneous, varying in size and location, with some patients having multiple *FLT3*‐ITDs. Bulk cell‐based approaches are limited in their ability to reveal the clonal structure in such cases. Using single‐cell proteogenomic sequencing (ScPGseq), we attempted to identify a relapse‐fated subclone in an AML case with mutations in *WT1*, *NPM1*, and *FLT3* tyrosine kinase domain and two *FLT3*‐ITDs (21 bp and 39 bp) (low AR) at presentation, then relapsed only with *WT1* and *NPM1* mutations and one *FLT3*‐ITD (high AR). This relapse‐fated subclone at presentation (∼2.1% of sequenced cells) was characterized by the presence of a homozygous 21 bp *FLT3*‐ITD resulting from copy neutral loss of heterozygosity (CN‐LOH) of chr13q and an aberrant, immature myeloid cell surface signature, contrast to the cell surface phenotype at presentation. In contrast to results from multicolor flow‐cytometry, ScPGseq not only enabled the early detection of rare relapse‐fated subclone showing immature myeloid signature but also highlighted the presence of homozygous 21 bp *FLT3*‐ITDs in the clone at presentation.

## 1 INTRODUCTION

Internal tandem duplication of the FMS‐like tyrosine kinase 3 (*FLT3*‐ITD) is one of the most common and clinically relevant mutations in acute myeloid leukemia (AML) [1, 2]. *FLT3*‐ITD is found in approximately 25%–30% of AML cases and often co‐occurs with *NPM1* (nucleophosmin 1) mutations [2, 3, 4]. Prior publications have commented on the importance of allelic ratio (AR), insertion size, location, and the number of Internal Tandem Duplications (ITDs) as being associated with diverse clinical outcomes [5, 6, 7, 8, 9, 10, 11, 12]. Based on these observations, the 2017 European LeukemiaNet (ELN) recommendations commented that *NPM1*‐mutated AML patients can be categorized into either intermediate or favorable risk groups depending on their *FLT3*‐ITD status and the AR [13]. It is recommended that patients with an *NPM1* mutation and *FLT3*‐ITD ≥0.5 receive allogeneic hematopoietic cell transplantation, while high dose consolidation chemotherapy is considered sufficient for patients with *FLT3*‐ITD AR <0.5 receiving curative‐intent chemotherapy.

As information regarding the presence or absence of *FLT3* mutations is required within days of diagnosis for the choice of proper treatment for AML, polymerase chain reaction (PCR) of bulk Deoxyribonucleic Acid (DNA) or Ribonucleic Acid (RNA) is employed in diagnostic laboratories [14]. This approach can determine the size and AR of *FLT3*‐ITDs but does not provide information with regard to what is happening within individual cells. An AR of ≥0.5 means that in a significant proportion of cells, there has likely been a loss of the wild‐type *FLT3* allele, such that some cells contain only the ITD form of *FLT3*. This could occur due to loss of heterozygosity (LOH), or reduction to homozygosity, at the *FLT3* locus. Such cells have a very high probability of causing relapse in the absence of allogeneic HCT [15, 16, 17, 18]. However, when the AR is <0.5, there is uncertainty about the nature of the *FLT3*‐ITD carrying leukemic population. As most diagnostic laboratories use DNA from bulk peripheral blood or bone marrow nucleated cells, a spuriously low AR of <0.5 can come about if there is significant contamination of the sample by residual nonleukemic cells. It is also possible to miss cells with only the *FLT3*‐ITD form of *FLT3* if the *FLT3*‐ITD occurred late in disease development, as is often the case, and is subclonal at the time of assessment. Finally, the bulk assessment does not inform whether the mutations in cases with several *FLT3* isoforms are present in a single clone of cells or come about because of multiple clones.

While bulk methods cannot resolve questions of co‐occurrence of mutations in a cell or identify subclones that have lost the wild‐type allele *FLT3*, single‐cell‐based approaches can overcome these limitations and provide an opportunity to capture subclonal genetic events. Studies utilizing single‐cell sequencing have generated clinically and biologically relevant information in AML including the pattern of acquisition of mutations and clonal evolution, as well as deconvolution of bulk AML samples based on surface markers and mutations [19, 20, 21, 22, 23, 24, 25, 26, 27]. With longitudinal samples, emerging mutation patterns post‐*FLT3* inhibitor treatment have also been observed [23].

In this report, we describe our investigation of the leukemic cells of a patient with AML using single‐cell proteogenomic sequencing (ScPGseq) allowing for simultaneous determination of DNA mutations and cell surface proteins at the single‐cell level. Through this approach, we demonstrate that it is possible to accurately characterize multiple *FLT3*‐ITDs at the single‐cell level. More importantly, by integrating DNA mutation and cell surface phenotypes, we show that a preexisting relapse‐fated subclone could be identified at the time of initial diagnosis.

## 2 MATERIALS AND METHODS

### 2.1 Single‐cell proteogenomic sequencing

Cryopreserved peripheral blood mononuclear cells at initial diagnosis and relapse were obtained from a 46‐year‐old female who was diagnosed with de novo AML; these were used for quantitative Polymerase Chain Reaction (qPCR) for identification of *FLT3*‐ITD mutations, targeted bulk DNA sequencing and ScPGseq. ScPGseq was performed using the Mission Bio's AML panel and 16 barcoded oligonucleotide‐conjugated antibodies following the manufacturer's protocols (Table S1).

Using ScPGseq, we identified the mutation profile and abundance of 16 cell surface markers (Table S2). Detailed procedures on data filtering (Table S3), *FLT3*‐ITD detection, Single Nucleotide Polymorphism (SNP) array analysis, and protein abundance analyses are described in the supplementary appendix. This study was approved by the institutional ethics review board at Princess Margaret Cancer Centre and was conducted following the Declaration of Helsinki. All sequencing data used in this study have been deposited to European Nucleotide Archive (accession number: PRJEB46675)

### 2.2 Statistical analysis

All statistical analyses were performed using the R programming language (R Foundation for Statistical Computing) [28]. To compare discrete and continuous variables, Fisher's exact test and Student's *t*‐test or Mann–Whitney *U* test were used accordingly. Bonferroni correction was used to adjust for multiple comparisons [29].

## 3 RESULTS

### 3.1 Description of clinical testings and study subject

**FIGURE 1:**
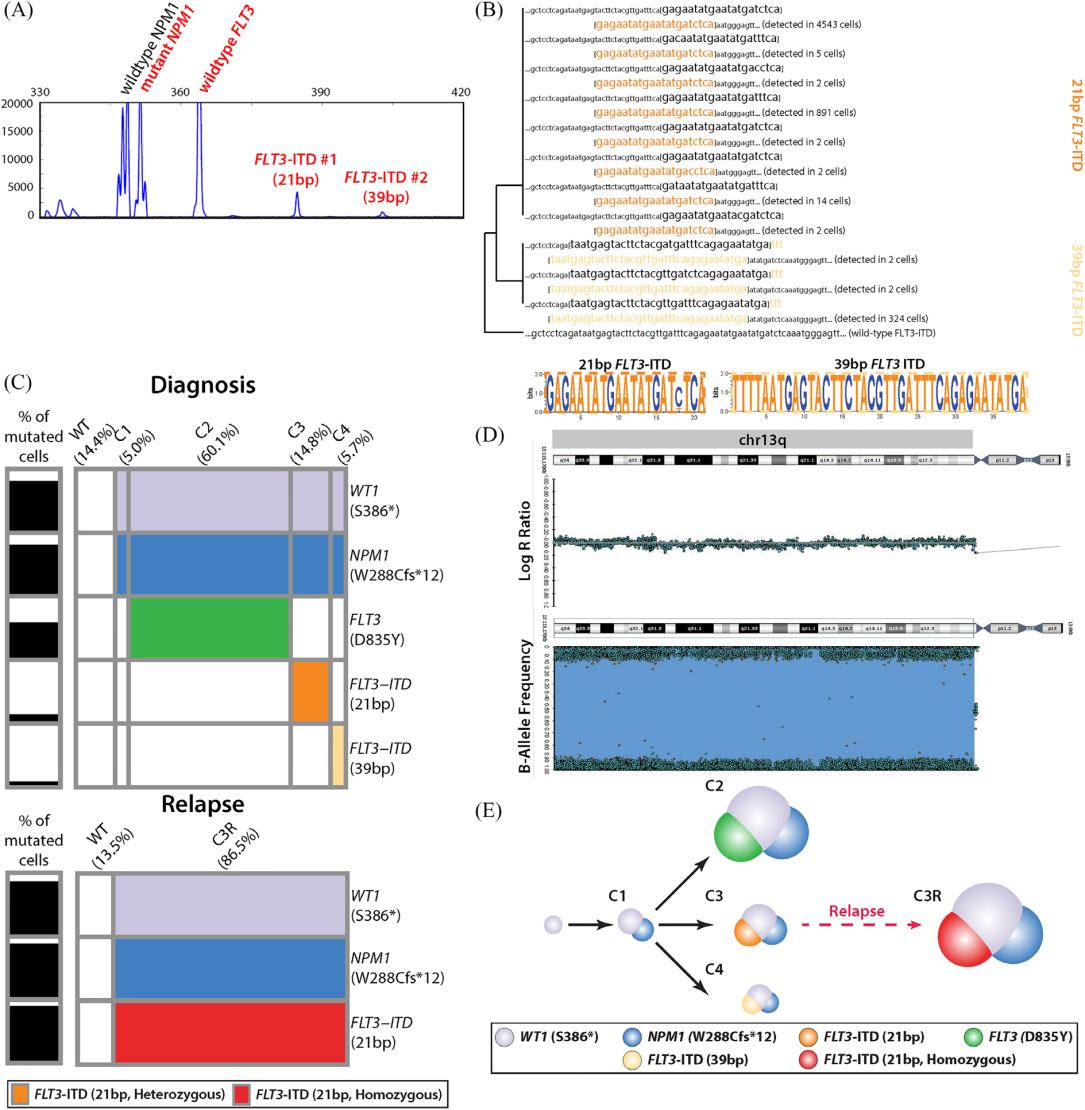
Analyses of DNA mutations from diagnosis to relapse. (A) Fragment analyses identified two ITDs in the *FLT3* gene (one at 21 bp and another at 39 bp). (B) Tree‐based analysis of two different *FLT3*‐ITDs identified in single‐cell sequencing data. (C) Two heatmaps describing clonal architectures at diagnosis and relapse. Each row indicates five and three mutations identified in the diagnosis and relapse samples. Each column indicates each subclone. The color of each cell describes mutation status except gray and white, which both indicate the absence of mutations. (D) SNP array identifies copy‐neutral loss of heterozygosity (CN‐LOH) event at chromosome 13q in the relapse sample. The log R ratio plot on the top indicates there is no copy number change (i.e., two copies, top plot) whereas the pattern of B‐allele frequency (bottom plot) shows the presence of only a major allele (A allele) or minor allele (B allele) at each SNP location distal to chromosome band 13q12.1. A normal heterozygous SNP profile is seen only directly adjacent to the centromere (13q centromere → 13q12.1). (E) Schematic view of clonal architecture at each sampling time point and evolution model using the only pattern of DNA mutations

Cells were obtained from a 46‐year‐old female with de novo AML at the time of initial diagnosis and relapse (Table S4). Using bulk RNA and a qPCR‐restriction fragment length polymorphism (RFLP) assay, two *FLT3*‐ITDs (#1. size: 21 bp, level: 13% and #2. size: 39 bp, level: 3.5%) were identified at diagnosis (Figure 1A). The presence of these ITDs was confirmed using a bulk DNA sequencing assay (21 bp with a Variant Allele Frequency (VAF) of 8.8% and 39 bp with a VAF of 2.8%). In addition, the bulk DNA sequencing revealed a *FLT3* tyrosine kinase domain (TKD) mutation (D835Y at a VAF of 34.7%) and mutations in *NPM1* (W288Cfs*12 at a VAF of 41.4%) and *WT1* (Wilms’ tumor 1) (S386* at a VAF of 43.4%). Cytogenetics by G‐banding showed a normal karyotype (46, XX [24/24]). According to the 2017 ELN risk stratification, the case was classified into the favorable‐risk group based on *FLT3*‐ITD (low AR) and an *NPM1* (W288 hotspot) mutation.

At relapse, qPCR‐RFLP detected both the 21 bp *FLT3*‐ITD (level: 91.37%) and the *NPM1* mutation; the 39 bp *FLT3*‐ITD and *FLT3* D835Y were not detected. In concordance, bulk DNA sequencing revealed VAFs of 88.4% for the 21 bp *FLT3*‐ITD, 36.0% for *NPM1*, and 45.0% for *WT1* mutations. The 39 bp *FLT3*‐ITD and *FLT3* D835Y were not detected. Detailed description on immunophenotypes and course of treatment (Figure S1) are described in the supplementary appendix.

### 3.2 Clonal analyses of single cells detect three AML subclones with three distinct *FLT3* mutations at diagnosis and one AML clone at relapse

In the analysis of 2367 cells from the diagnostic sample, mutations were detected in *WT1* S386*, *NPM1* W288Cfs*12, *FLT3* D835Y, 21 bp *FLT3*‐ITD, and 39 bp *FLT3*‐ITD (Figure 1B,C, Figure S2 and S3A). After removing rare clones and potential false‐positive clones resulting from allelic dropout or multiplets, five populations were identified in 1942 cells (Figure 1C and Figure S4). The dominant clone, accounting for 60.1% of cells, contained the *WT1*, *NPM1*, and *FLT3* D835Y mutations (C2, 60.1%, 1,167/1,942 cells). Approximately, 5% of cells (5.0%, 97/1942 cells) carried only *WT1* and *NPM1* mutations. We consider this clone to be antecedent to C2 and refer to it as clone 1 (C1). There were two further clones, which in addition to having mutations of *WT1* and *NPM1* had either a 21 bp (14.8%, 287/1942 cells) or 39 bp (5.7%, 111/1942) *FLT3*‐ITD; these are referred to as C3 and C4, respectively. The remaining 14.4% of cells (Wild Type (WT) cells, 280/1942 cells) did not carry any of the five considered mutations.

The clonal analysis of 2226 cells in the relapse sample revealed two populations (Figure 1C and Figure S4). The largest fraction accounting for 86.5% (1925/2226 cells) carried *WT1* S386*, *NPM1* W288Cfs*12, and 21 bp *FLT3*‐ITD (Figure 1B,C, Figure S2 and S3B). In contrast to C3 (*WT1* ^+^/*NPM1* ^+^/21 bp *FLT3*‐ITD^+^ cells at initial diagnosis), there was no wild‐type *FLT3* allele present in these cells (median single‐cell VAF [scVAF] = 100%). We refer to this clone as C3R as it most likely arose from C3 by loss of the wild‐type *FLT3* allele (Figure 1C and Figure S5). The remaining 301 cells (13.5%, 301/2226 cells) did not carry any mutations. An interesting feature of the C3R cells was that only the 21 bp *FLT3*‐ITD and not the wild‐type *FLT3* allele was detected (Figure S4A), suggesting reduction to homozygosity at the *FLT3* locus. As can be seen in Figure 1D, the loss and duplication of chromosome 13q was confirmed using SNP array analysis.

By incorporating clonal architectures at diagnosis and relapse, we inferred clonal evolution from diagnosis to relapse (Figure 1E). Based on the mutation pattern (Table S5), we inferred that the *WT1* mutation occurred first, followed by the acquisition of the *NPM1* mutation. Subsequently, three *FLT3* mutations developed as individual events in unique C1 cells, establishing three distinct subclones. Following treatment and relapse, only the subclone with the 21 bp *FLT3*‐ITD of C3 was found, but as noted above C3R had lost the normal *FLT3* allele (Figure S5A).

### 3.3 Cell surface phenotype identifies a subclone at diagnosis, which is dominant in subsequent AML relapse

**FIGURE 2:**
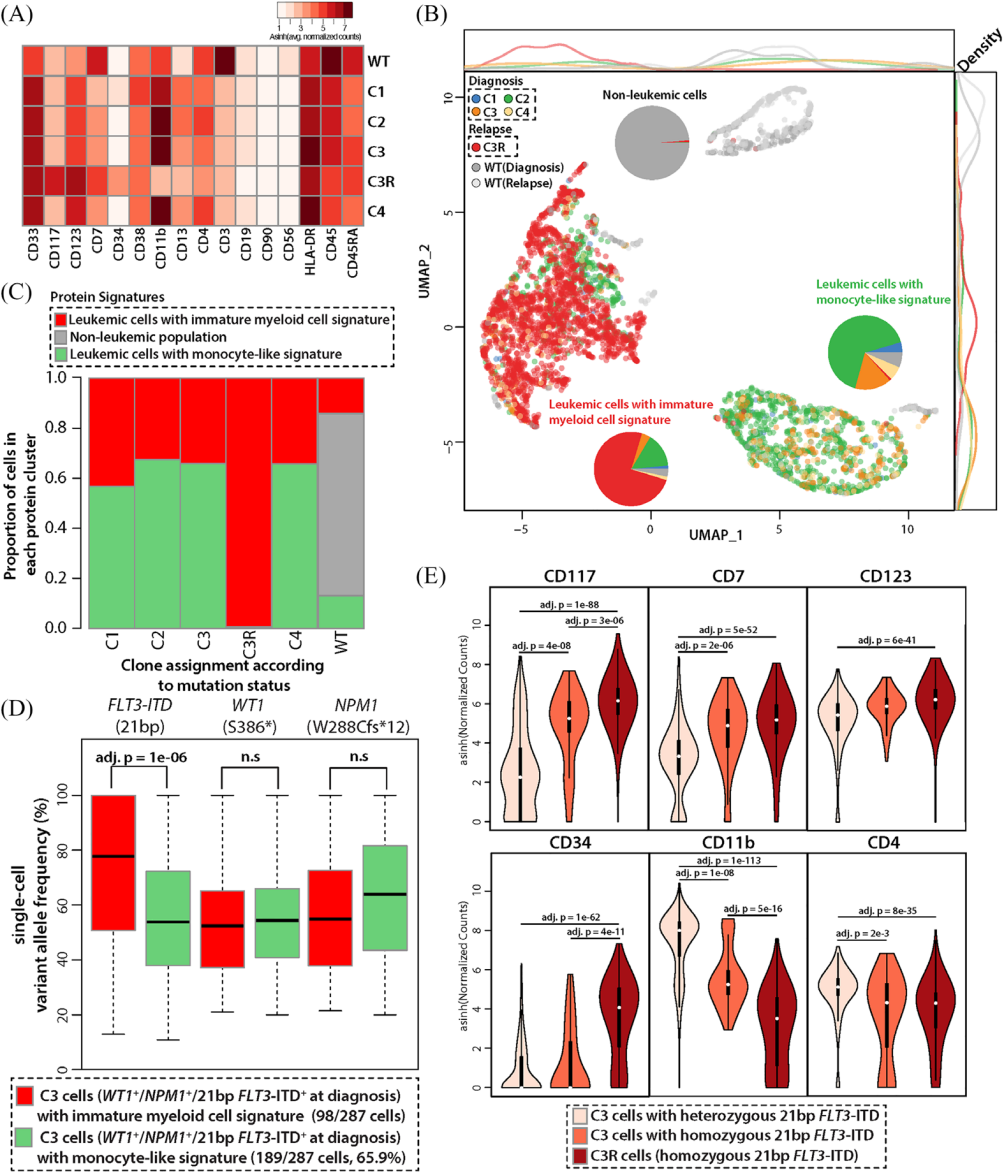
Profile and dynamics of cell surface proteins. (A) The expression level of 16 cell surface proteins for each population. Color intensity represents an average expression of each protein (asinh‐transformed), where white indicates no expression and red indicates high expression. (B) Uniform manifold approximation and projection (UMAP) analysis identified three major clusters of cells according to protein expressions. The first cluster mostly consists of nonleukemic cells from both diagnosis and relapse samples (top right). The second cluster of cells represents cells from diagnosis cells (bottom right). Lastly and interestingly, the third cluster on the left side of the plot consists of cells from both relapse and diagnosis cells. Three pie charts describe the proportion of each subclone in each of the cell clusters. (C) The proportion of cells in each protein‐based cell signature according to clone assignment based on DNA mutation status. (D) Proportion of reads supporting 21 bp *FLT3*‐ITD, *NPM1*, and *WT1* mutations in cells in the C3 clone according to cell surface phenotypes. Among 287 cells in C3, 98 cells were showing stem cell‐like signature (red), and 189 cells were showing monocyte‐like signature (green). (E) Expression of six cell‐surface proteins (CD117, CD7, CD123, CD34, CD11b, and CD4) among two subsets of C3 cells according to zygosity of 21 bp *FLT3*‐ITD and C3R cells (homozygous)

We analyzed the presence and distribution of 16 cell surface proteins within C1‐4, C3R, and WT cells. Noticeably, we found that the expression of cell surface proteins differed greatly between WT and mutant cells (Figure 2A, Figures S6 and S7). Compared to mutant cells, the WT cells were enriched for cells with high expression levels of CD3, CD45, and CD56, and lower expression levels of CD123 and CD33, indicating the WT cells to be predominantly lymphocytes or cells of nonmyeloid phenotype. In contrast, and in keeping with the diagnosis of AML, the mutation‐bearing cells all expressed myeloid antigens. Dimension reduction of 16 protein expressions using uniform manifold approximation and projection identified three major clusters of cells including one cluster nearly exclusive to WT cells (428/4168 cells [10.3%], “nonleukemic”) (Figure 2B) [30]; the WT cells were predominantly T cells. Two other clusters, consisting of almost all mutant cells showed distinct protein expression profiles. One cluster showed higher expressions of CD117 and CD34, which we termed, “leukemic cells with immature myeloid cell signature” (2551/4168 cells [61.2%]). The second cluster had a high expression of CD11b; we refer to this subset as, “leukemic cells with monocyte‐like signature” (1189/4168 cells, [28.5%]) (adjusted *p* < 1e‐08 for all three protein expression levels). For each of C1‐4, the proportion of cells with the immature phenotype was about 1/3 of the population (Figure 2C). In contrast, nearly all cells in C3R showed an immature myeloid cell signature (99.3%, 1912/1925 cells) with high levels of CD117, CD123, and CD34 (Figure 2A, Figures S6 and S7). In addition, there was aberrant high expression of CD7 and reduced expression of CD11b.

### 3.4 Integration of cell surface phenotype with DNA mutation profiles refines the model of clonal evolution

We integrated mutation profiles with cell surface protein signatures and compared scVAFs and zygosity in the four leukemic clones present at diagnosis. In doing this, we wanted to determine if there was a difference in the mutation profile of two cell surface signatures within a clone. We were also interested in determining if it would be possible to identify homozygous ITD‐positive (ITD^Hom^) cells (scVAF ≥95% as a cut‐off). The distribution of the mutations was similar in the immature and mature population of cells; however, this was not the case for 21 bp *FLT3*‐ITD. C3 consists of 287 cells; 98 and 189 cells (34.1% and 65.9%) having immature and monocyte‐like cell surface signatures, respectively. Within C3, cells with immature myeloid cell signature had higher scVAFs of the 21 bp *FLT3*‐ITD when compared to monocyte‐like C3 cells (Figure 2D, mean 73% vs. 56%, adjusted *p*‐value = 1.0e‐6). In line with higher scVAFs, 41 of the 98 (42%) immature cells were homozygous for the 21 bp *FLT3*‐ITD, while only 11 of the 189 (5.8%) monocyte‐like cells were homozygous for *FLT3*‐ITD (Figure S8, *p*‐value = 2.7e‐13). Taken together, a proportion of the immature cells in C3 are truly homozygous for the 21 bp *FLT3*‐ITD (i.e., C3‐ITD^Hom^). It is of note that C3‐ITD^Hom^ cells had a similar cell surface phenotype as observed in C3R cells, with high expression levels of CD7, CD33, CD117, and CD123 and decreased levels of CD11b and CD4 (Figure 2E and Figure S9).

**FIGURE 3:**
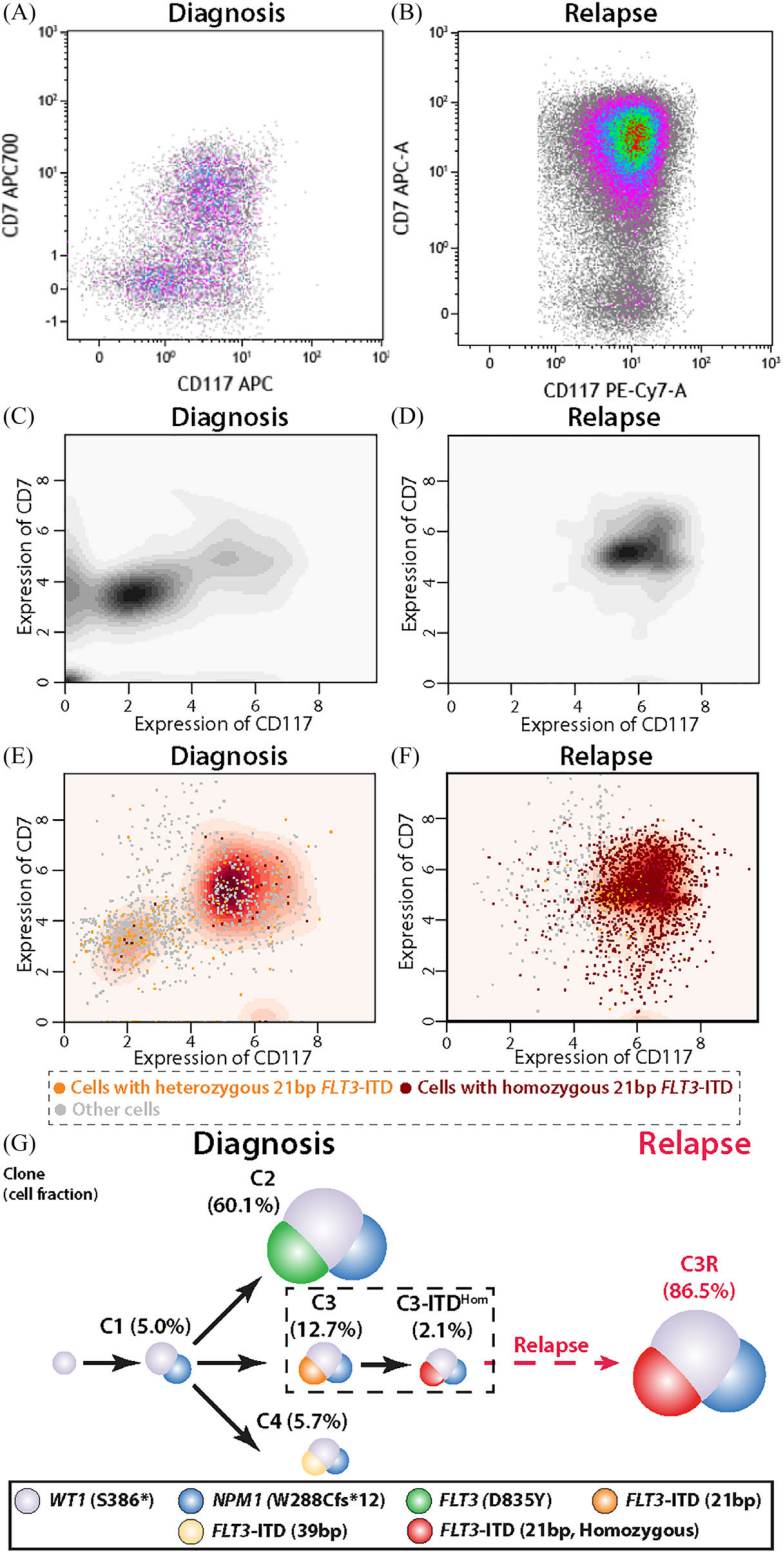
Enrichment of homozygous 21 bp *FLT3*‐ITD in aberrant leukemic cells via flow cytometry and single‐cell proteogenomic sequencing and refinement of DNA‐based clonal model using cell surface phenotypes. (A) Distribution of cells according to CD117 and CD7 expression at (A) presentation and (B) relapse via flow cytometry. 2D kernel density plot describes the density of all cells from the (C) diagnosis sample and (D) relapse sample. In (E) and (F), cells with 21 bp *FLT3*‐ITD (i.e., C3 or C3R cells) are either colored orange (in case of heterozygous) or red (in case of homozygous). 2D kernel density in the background describes the density of cells with homozygous 21 bp *FLT3*‐ITD. As can be seen in (C) and (E), there is a significant increase of CD117 and CD7 expression in cells with homozygous 21 bp *FLT3*‐ITD compared to all cells. On the other hand, such shifts in CD117 and CD7 expression were not observed in the relapse sample. Expression levels in (C)–(E) are asinh transformed value of normalized counts. (G) Schematic view of clonal architecture at each sampling time point and evolution model after refining the DNA mutation‐based clonal evolution model using cell surface phenotype. Mainly, C3 cells in the DNA‐only model (287/1,942 cells, 14.8%) were further separated into C3 (246/1,942 cells, 12.7%) and C3‐ITD^Hom^ (41/1,942 cells, 2.1%)

To further confirm the presence of ITD^Hom^ cells, we compared results from ScPGseq with flow cytometry (Figure 3). Based on flow cytometry, we were able to observe two populations of AML cells at diagnosis according to expressions of CD117 and CD7 (Figure 3A). On the other hand, we observed a single population with high CD117 and CD7 expressions at relapse (Figure 3B). We performed the same analysis using results from ScPGseq, where we plotted cells from both samples according to their CD117 and CD7 expression levels (Figure 3C‐F). In line with the result from flow cytometry, we observed two populations of AML cells at diagnosis, but AML cells with immature myeloid cell signature were retained at relapse (Figure 3C,D). When focusing only on ITD^Hom^ cells (Figure 3E,F, indicated as red dots), there was a dramatic shift of high‐density region in the diagnosis sample, where most cells had low CD117 and CD7 expressions (i.e., monocyte‐like signature, Figure 3C) while ITD^Hom^ cells were enriched in the area of high CD117 and CD7 (i.e., immature myeloid cell signature, Figure 3E). We did not observe such a shift in the relapse sample (from Figure 3D–F).

Combining these results, cell surface phenotype complements and refines the model of clonal evolution inferred solely from patterns of DNA mutations (Figure 3G). Instead of C3 cells acquiring Copy Neutral Loss Of Heterozygosity (CN‐LOH) in chromosome 13q during relapse, C3‐ITD^Hom^ cells with immature myeloid cell signature were already present at very low frequency from presentation, survived or escaped from treatment, and became the dominant clone at relapse.

## 4 DISCUSSION

The current study utilized ScPGseq to characterize multiple *FLT3*‐ITDs at high resolution and leveraged the methodology to identify the relapse‐fated subclone at the time of AML presentation. Miles et al. and Morita et al. have used ScPGseq to evaluate a variety of AML cases and showed the value of the methodology in assessing clonal architecture based on recurrent mutations and cell surface protein expression [24, 25]. Here, in this study, we have further expanded on the utility of ScPGseq to understand the AML development and relapse in a single case of AML, that at presentation had been assigned to the ELN favorable‐risk group based on the presence of *NPM1* mutation and the low *FLT3*‐ITD AR at presentation. Our in‐depth analyses of paired samples obtained at times of initial diagnosis and relapse revealed underlying dynamics of genetic/immunophenotypic diversity in addition to resultant clonal architectures. We observed therapeutic intervention including midostaurin decimated most AML clones including the dominant AML clone at presentation (i.e., C2, *WT1^+^*/*NPM1^+^*/*FLT3*‐TKD^+^) but provided potent selective pressure for the survival and expansion of a relapse‐fated subset of C3, C3‐ITD^Hom^. They were enriched for the homozygous 21 bp *FLT3*‐ITD, resulting from CN‐LOH of chromosome 13q (i.e., ITD^Hom^) and had an immunophenotype distinct from the rest of C3 cells (Figure 3G). It was not possible to define this subclone with bulk cell sequencing, SNP‐arry, or multi‐color flow cytometry. However, ScPGseq enabled the detection of a relapse‐fated subclone at the time of initial diagnosis and provides an understanding of the relapse in this patient, which is not infrequently seen in AML cases with low *FLT3*‐ITD AR. Our observation of altered phenotype in the C3R cells raises the potential of considering aberrant cell surface phenotypes in risk stratification. In order to reach a clearer conclusion on prognostic relevance of ITD^Hom^ cells, future study is strongly warranted in a larger AML cohort of patients with FLT3‐ITD AR.

Having identified CN‐LOH for the *FLT3*‐ITD in the relapse sample, we closely assessed *FLT3*‐ITDs in the presentation sample. There was a significant population within a subset of C3 cells (i.e., C3‐ITD^Hom^) with the loss of the wild‐type *FLT3* allele. Importantly, C3‐ITD^Hom^ had blocked the pattern of differentiation and aberrantly increased expression of CD7 as was found for C3R cells. As demonstrated here and previously by Stirewalt et al., using colony‐forming assay and single‐cell PCR, the homozygous *FLT3*‐ITD can be present at the time of diagnosis in AML patients with low *FLT3*‐ITD AR [31]. In the particular case presented here, a subset of C3 cells acquired CN‐LOH of chromosome 13q and thus created the equivalent of a ITD^Hom^ cell, which is presumably chemoresistant. The prevalence of ITD^Hom^ cells at presentation was 41 of 1942 cells (2.1%). Although it was possible to identify a characteristic cell surface protein expression pattern on these cells that included increased expression of CD34, CD117, and CD123, decreased expression of CD11b and, aberrant expression of CD7, it would be challenging to achieve sufficient enrichment in a routine diagnostic laboratory to identify the ITD^Hom^ cells. In addition, the increased expressions of CD7 and CD34 that we observed in this patient are not universal in AML patients that have a *FLT3*‐ITD and CN‐LOH of chromosome 13q as described by Soare et al. [32]. To truly identify a good risk group of patients with an *NPM1^+^* and low AR *FLT3*‐ITD, it would be of value to identify cell surface protein signatures that provide a means for identifying cells with CN‐LOH for chromosome 13q at the time of diagnosis. It should be noted that the current experiments cannot determine whether the shift in cell surface phenotype was due to CN‐LOH of the *FLT3*‐ITD, concomitant loss of other genes located in the chromosome 13q, or acquisition of other mutations or epigenetic changes not assessed in the current study. Based on our findings, we anticipate that further study will eventually help in enhancing clinical decision‐making for *FLT3*‐ITD^+^ AML cases.

## AUTHOR CONTRIBUTIONS

TaeHyung Kim, Mark D. Minden, and Dennis Dong Hwan Kim designed the study. Mark D. Minden provided samples and clinical data. Hyewon Lee, Gurbaksh Basi, and Troy Ketela performed single‐cell sequencing. Adam C. Smith performed SNP array analyses. Hyewon Lee, Jose‐Mario Capo‐Chichi, and Anne Tierens performed the pathologic analyses. TaeHyung Kim, Jose‐Mario Capo‐Chichi, and Zhaolei Zhang analyzed the sequencing data and performed computational analyses. TaeHyung Kim, Hyewon Lee, Jose‐Mario Capo‐Chichi, Myung Hee Chang, Young Seok Yoo, Adam C. Smith, Anne Tierens, Mark D. Minden, Zhaolei Zhang, and Dennis Dong Hwan Kim interpreted the data and results. TaeHyung Kim, Zhaolei Zhang, and Dennis Dong Hwan Kim performed statistical analyses. TaeHyung Kim, Mark D. Minden, and Dennis Dong Hwan Kim wrote the manuscript with inputs from all authors. Mark D. Minden and Dennis Dong Hwan Kim contributed equally as co‐senior authors. All authors read and approved the manuscript.

## CONFLICT OF INTEREST

The authors declare no competing financial interests.

## Supporting information

Figure S1

Figure S2

Figure S3

Figure S4

Figure S5

Figure S6

Figure S7

Figure S8

Figure S9

Supporting Information

Table S1

Table S2

Table S3

Table S4

Table S5
