# Supplementary figures and images for: Single cell proteogenomic sequencing identifies a relapse‐fated AML subclone carrying FLT3‐ITD with CN‐LOH at chr13q
Source: EJHaem. 2022 Feb 24;3(2):426–33. doi: 10.1002/jha2.390 (PMC9175792; doi:10.1002/jha2.390)

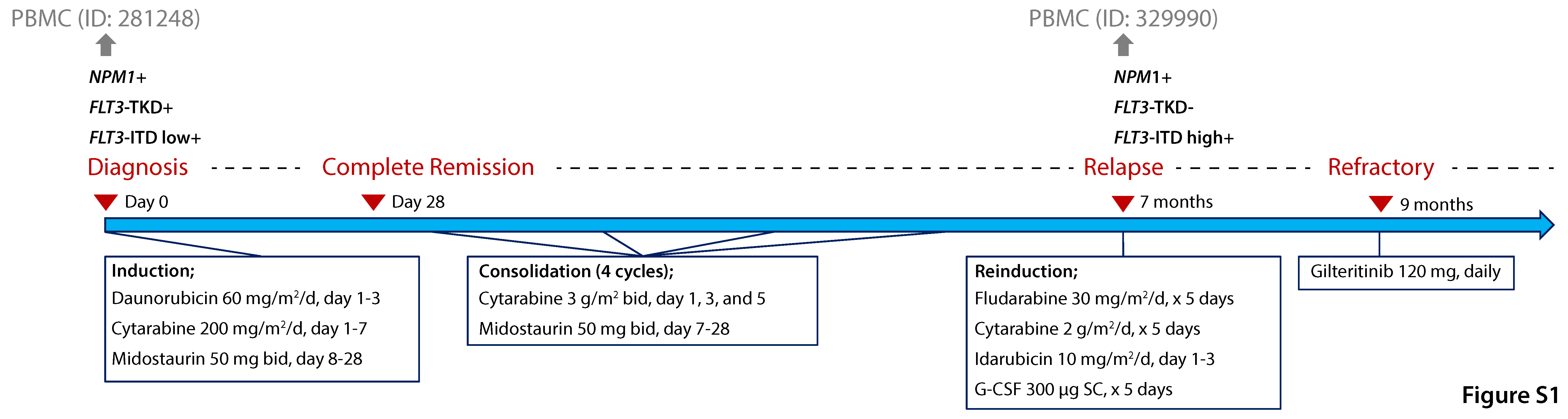

Supplement: Supplementary file 1 — Figure S1 [file JHA2-3-426-s004.tiff]

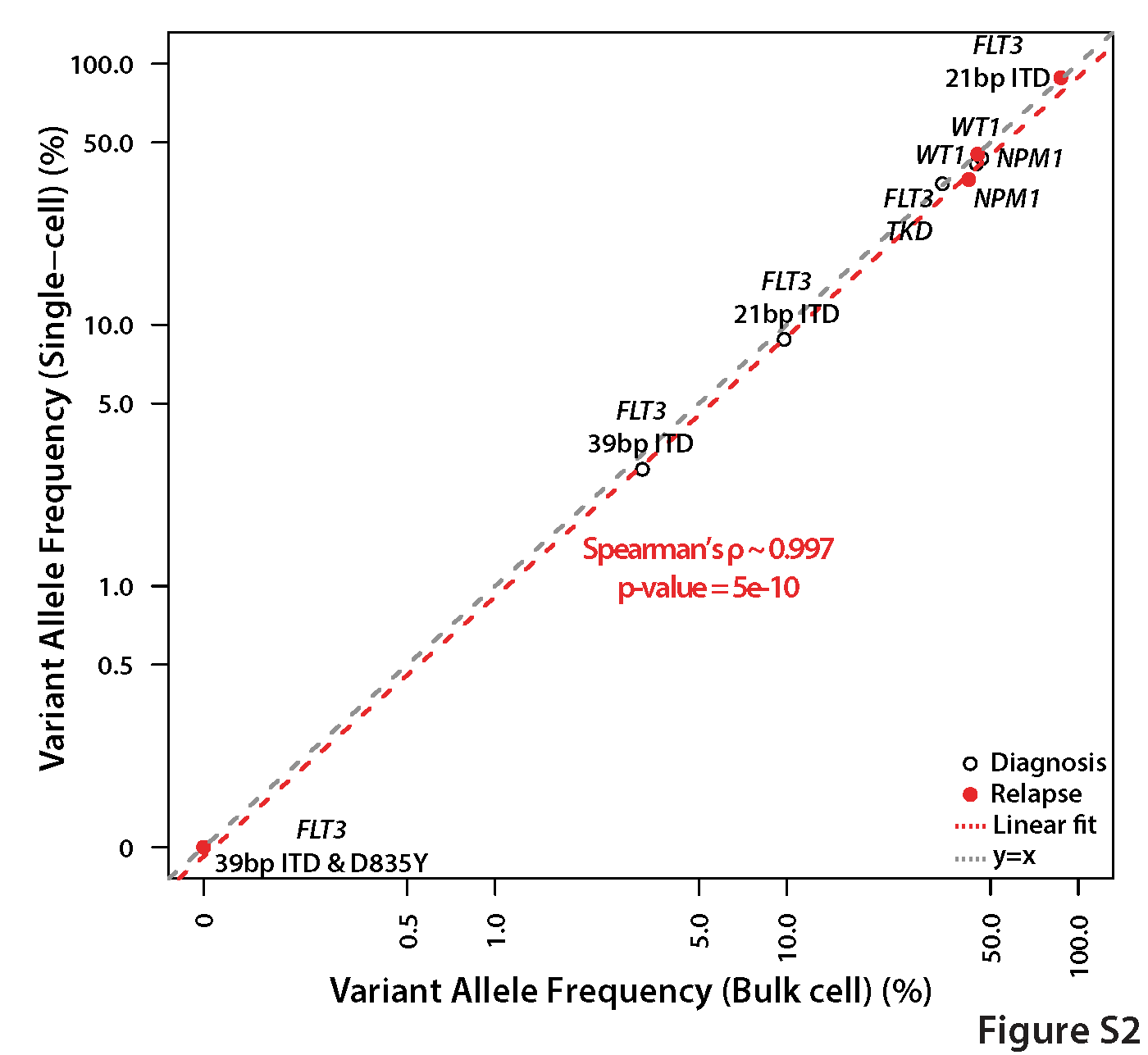

Supplement: Supplementary file 2 — Figure S2 [file JHA2-3-426-s011.tiff]

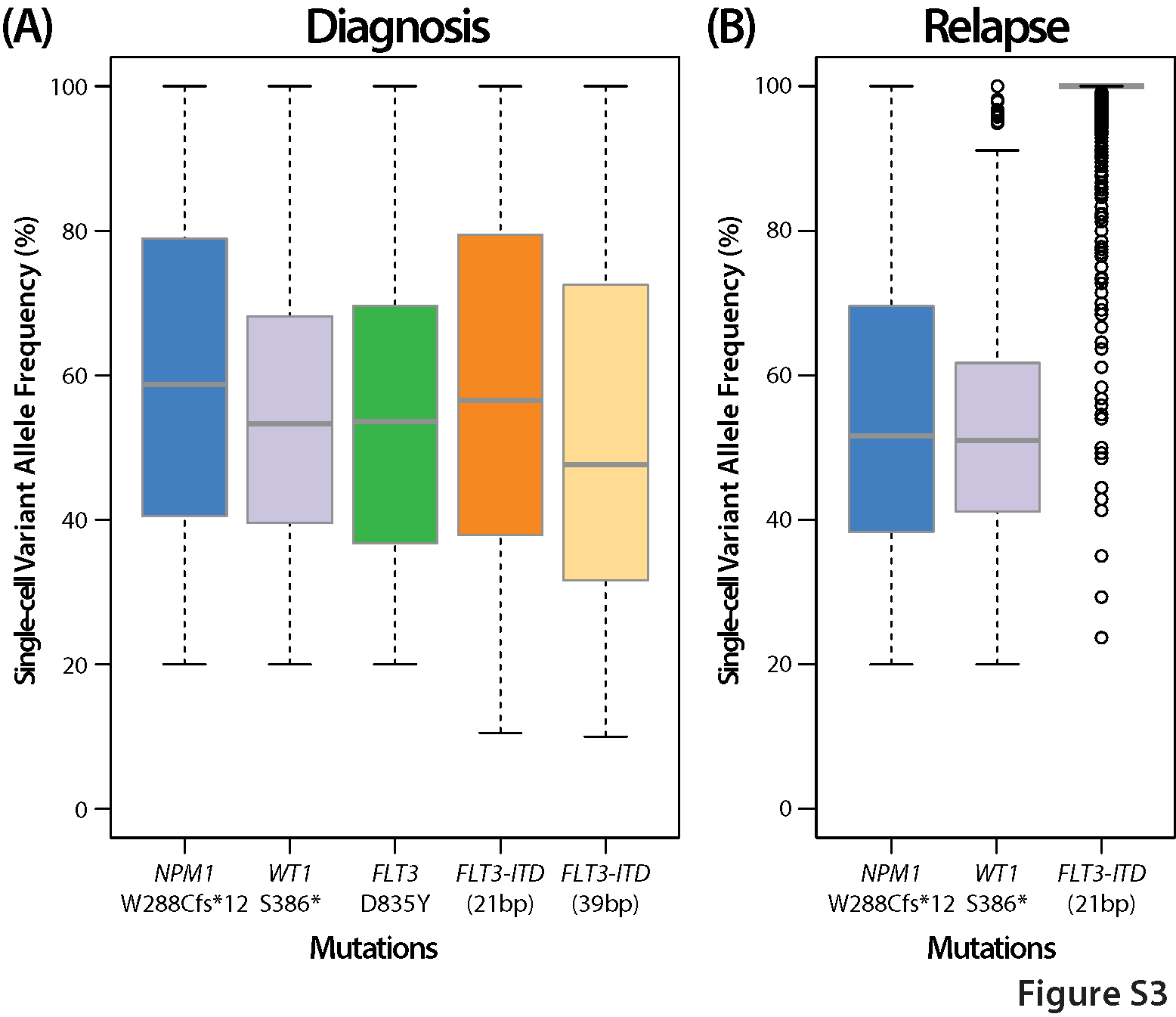

Supplement: Supplementary file 3 — Figure S3 [file JHA2-3-426-s001.tiff]

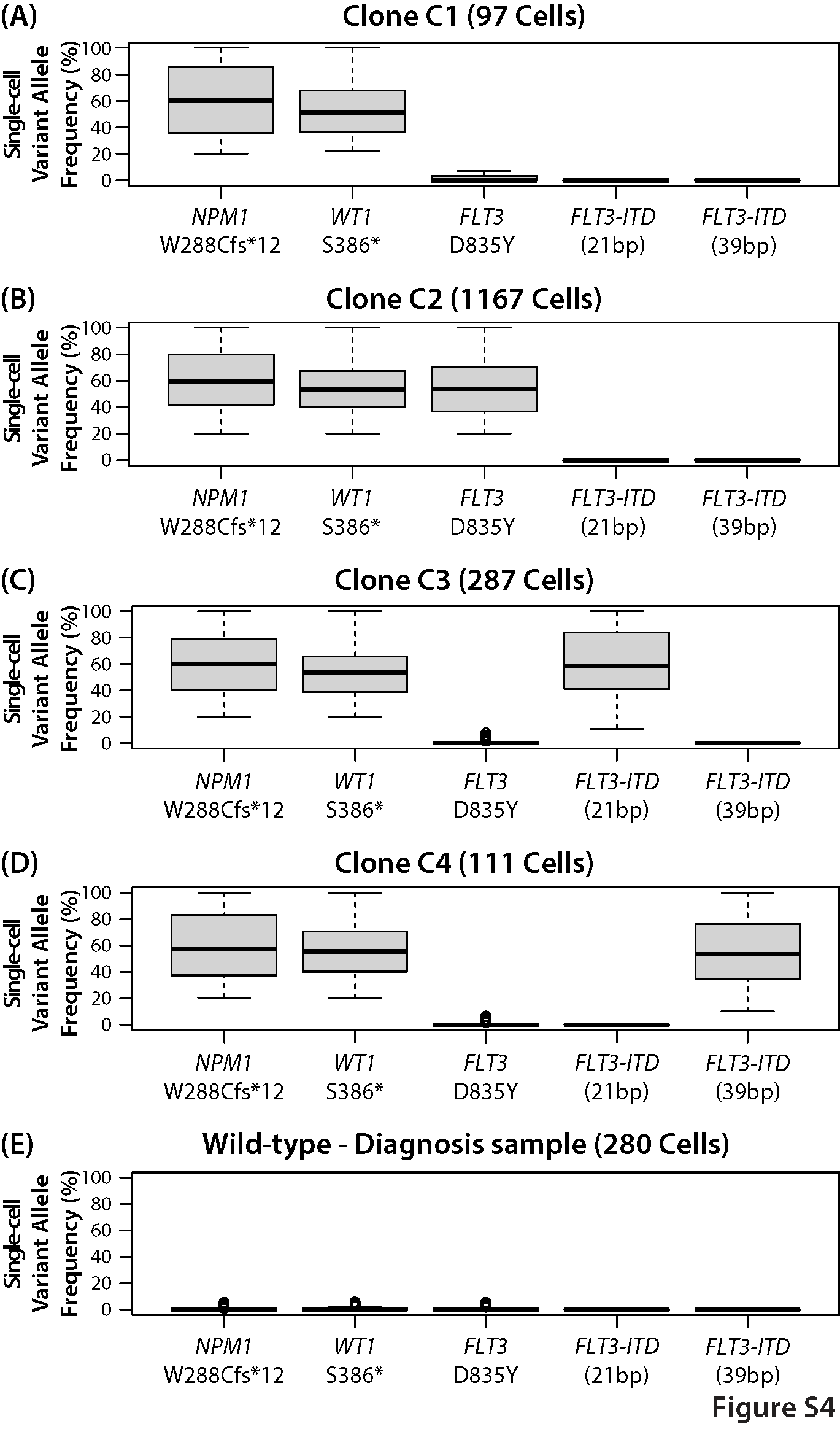

Supplement: Supplementary file 4 — Figure S4 [file JHA2-3-426-s014.tiff]

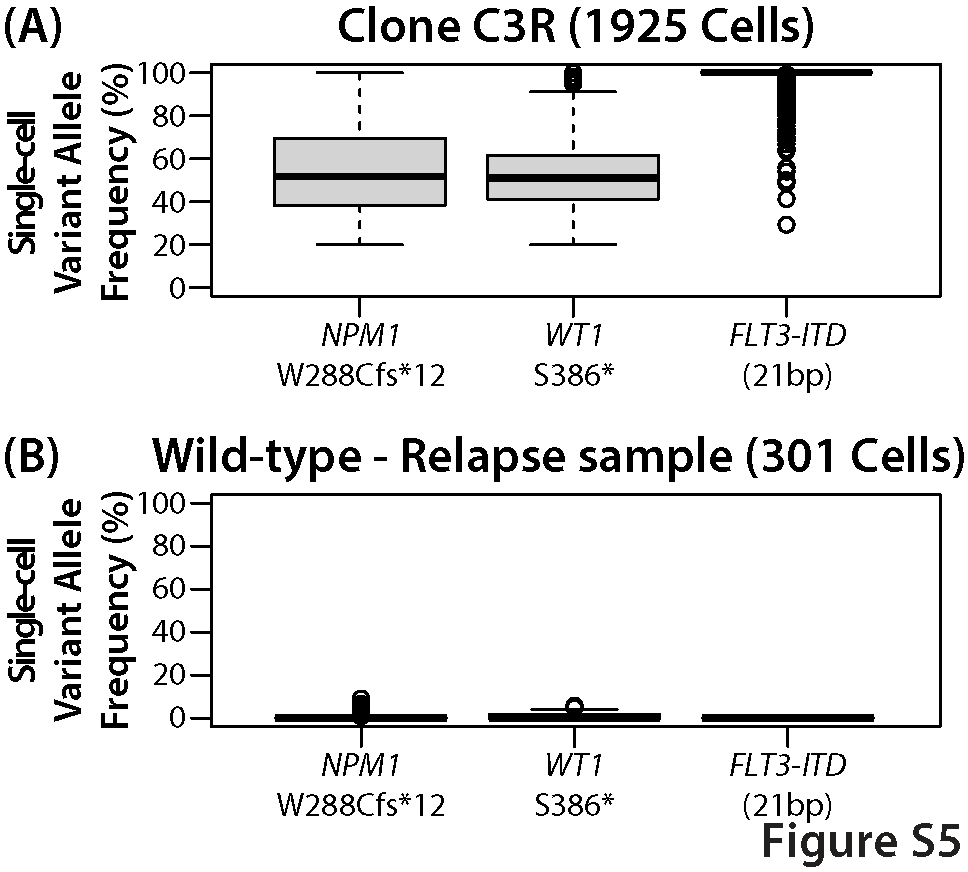

Supplement: Supplementary file 5 — Figure S5 [file JHA2-3-426-s006.tiff]

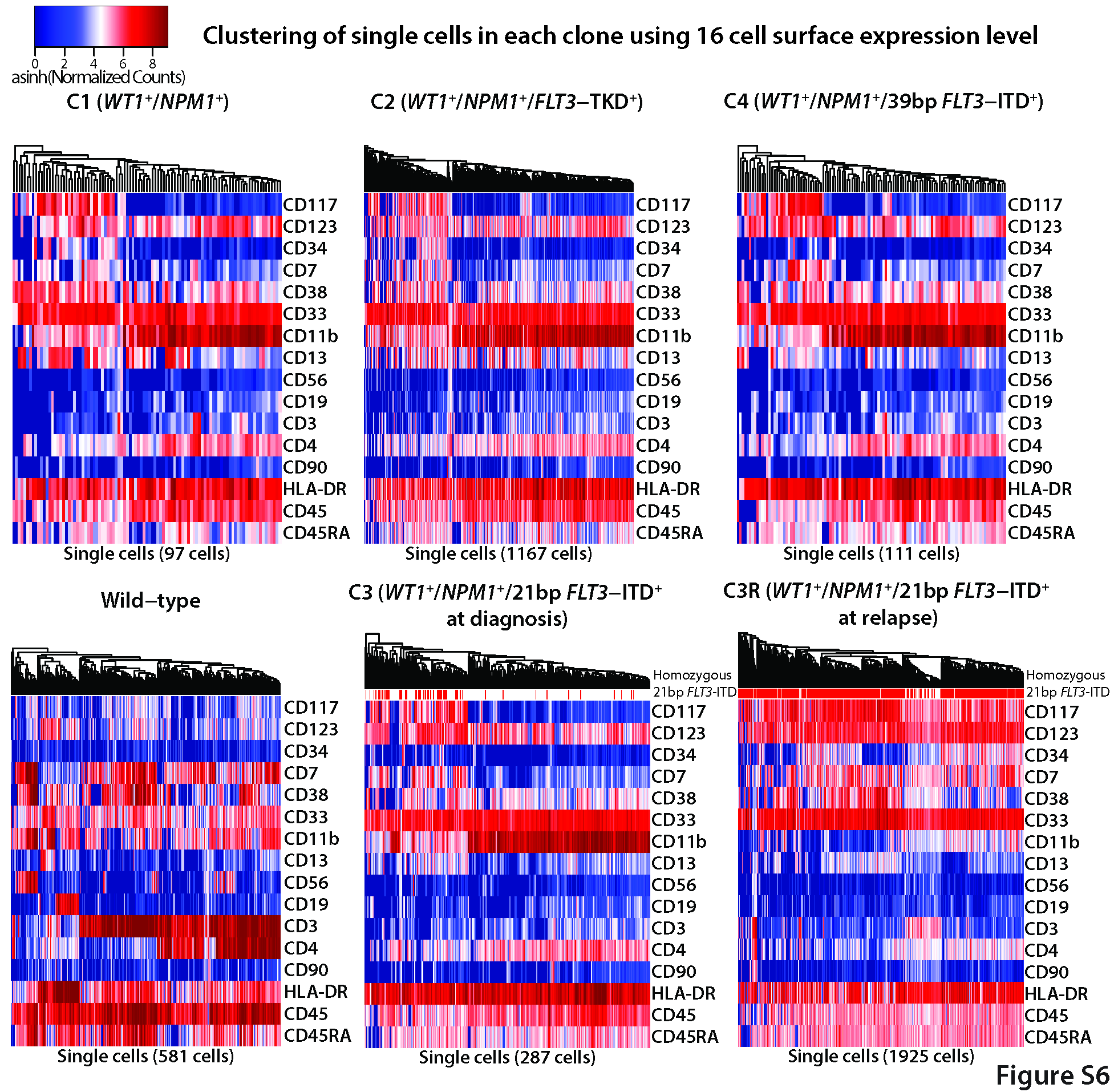

Supplement: Supplementary file 6 — Figure S6 [file JHA2-3-426-s012.tiff]

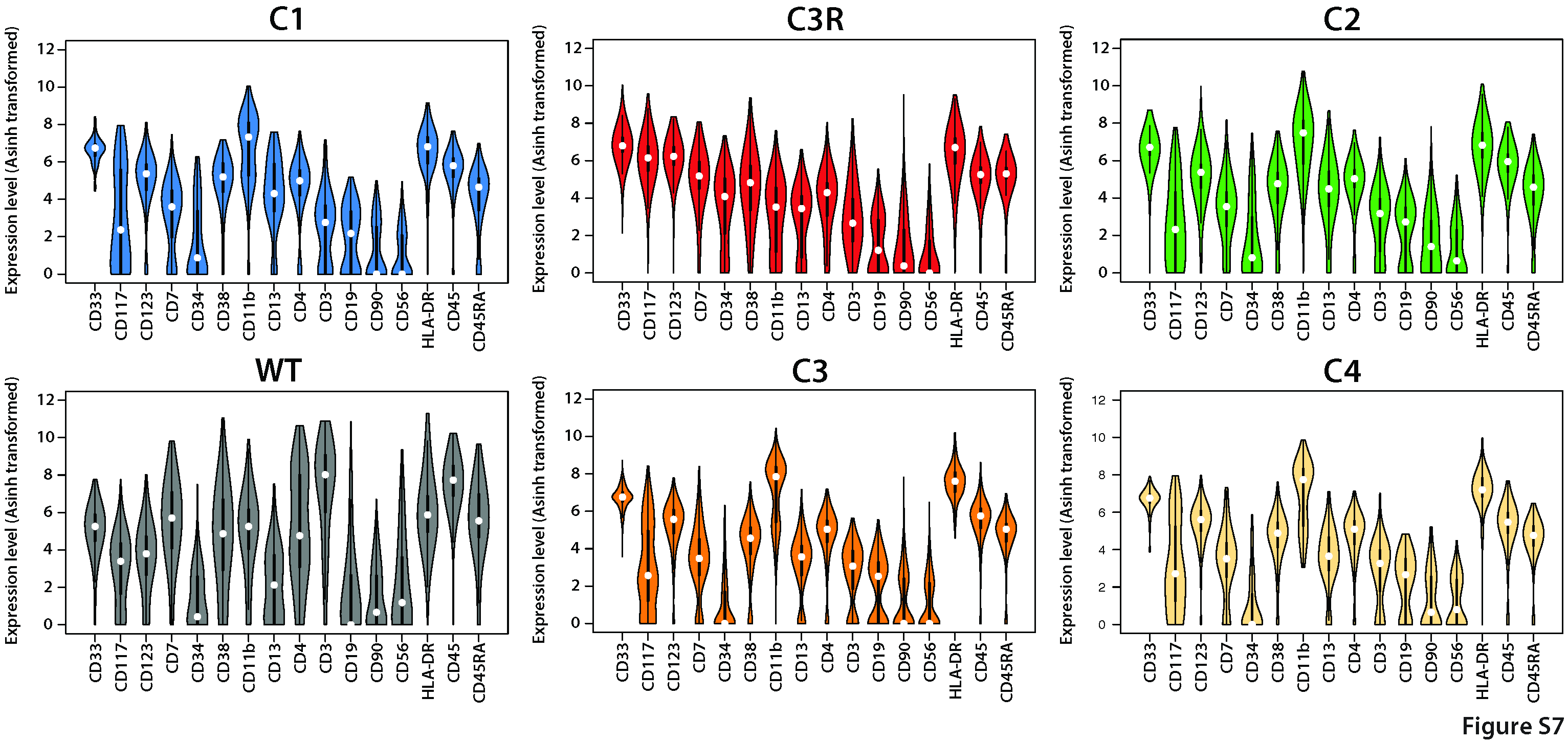

Supplement: Supplementary file 7 — Figure S7 [file JHA2-3-426-s002.tiff]

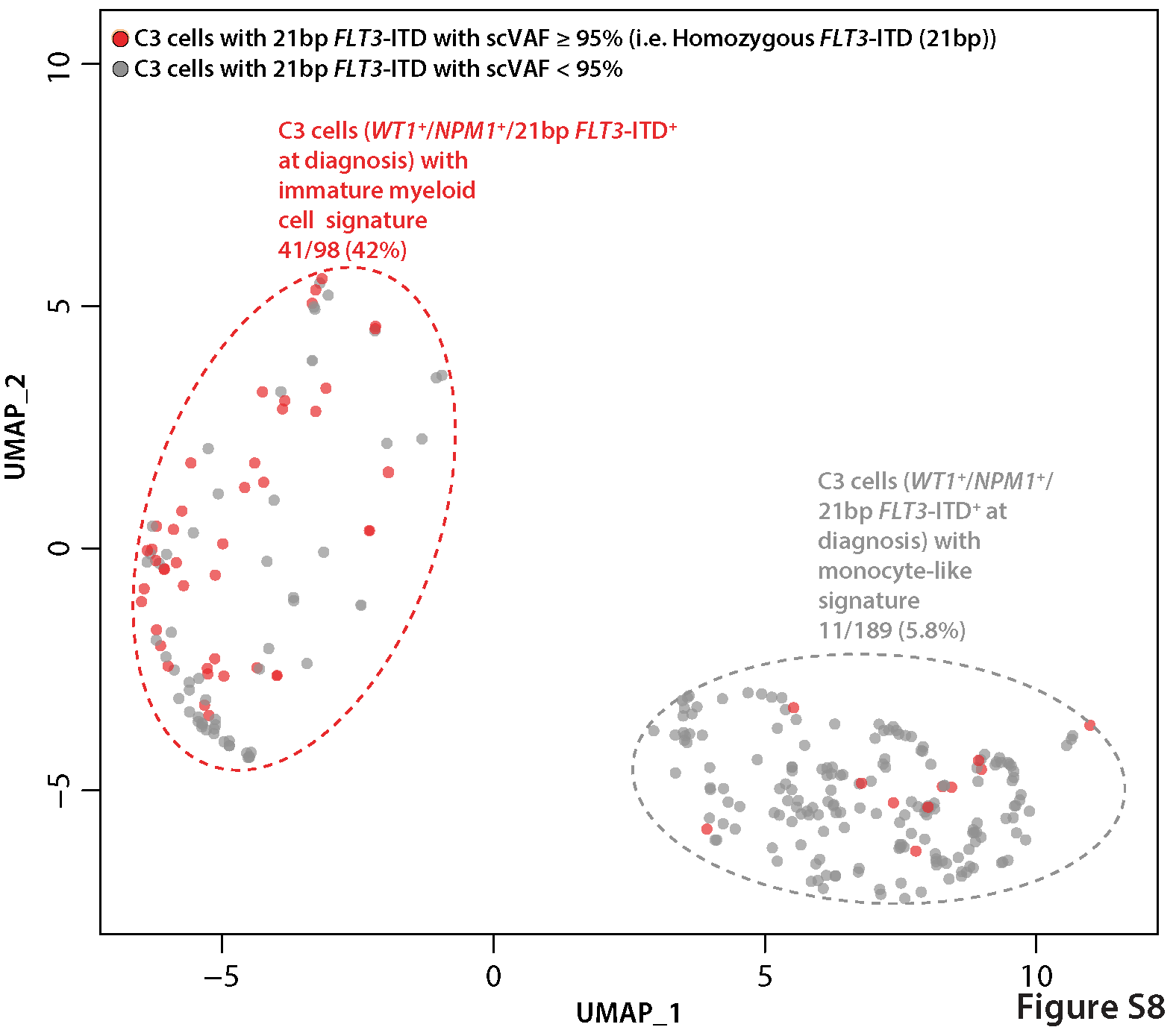

Supplement: Supplementary file 8 — Figure S8 [file JHA2-3-426-s010.tiff]

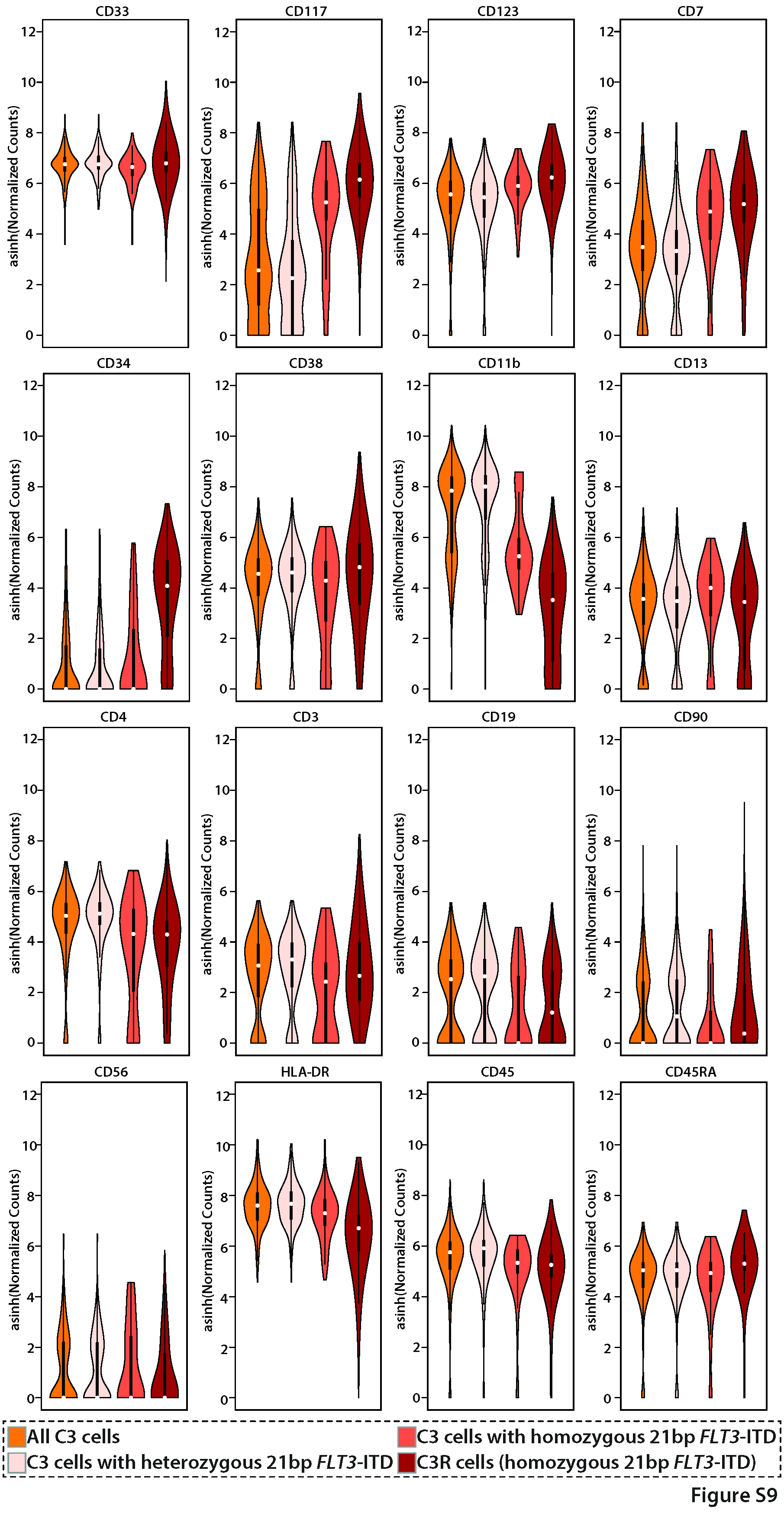

Supplement: Supplementary file 9 — Figure S9 [file JHA2-3-426-s008.tiff]
