# Supplementary material for: Single cell proteogenomic sequencing identifies a relapse‐fated AML subclone carrying FLT3‐ITD with CN‐LOH at chr13q
Source: EJHaem. 2022 Feb 24;3(2):426–33. doi: 10.1002/jha2.390 (PMC9175792; doi:10.1002/jha2.390)
Supplement: Supplementary file 10 — Supporting Information [file JHA2-3-426-s009.docx]

**Supplementary Information**

**This information has been provided by the authors to give readers detailed information about the study**

**Title: Single cell proteogenomic sequencing identifies a relapse-fated AML subclone carrying *FLT3*-ITD with CN-LOH at chr13q**

TaeHyung Kim^1,2,3^, Hyewon Lee^1,4^, Jose-Mario Capo-Chichi^5^, Myung Hee Chang^1,6^, Young Seok Yoo^1^, Gurbaksh Basi^7^, Troy Ketela^7^, Adam C. Smith^8,9^, Anne Tierens^8,9^, Zhaolei Zhang^2,3,10^, Mark D. Minden^1^, and Dennis Dong Hwan Kim^1,11^

^1^Division of Medical Oncology and Hematology, Princess Margaret Cancer Centre, Toronto, Ontario, Canada,

^2^Department of Computer Science, University of Toronto, Toronto, Ontario, Canada,

^3^The Donnelly Centre for Cellular and Biomolecular Research, University of Toronto, Toronto, Ontario, Canada,

^4^Center for Hematologic Malignancies, National Cancer Center, Goyang, Republic of Korea

^5^Department of Clinical Laboratory Genetics, Genome Diagnostics Laboratory Medicine Program, University of Toronto, Toronto, Ontario, Canada

^6^Division of Oncology-Hematology, Department of Internal Medicine, National Health Insurance Service Ilsan Hospital, Goyang, Republic of Korea.

^7^Princess Margaret Genomics Centre, Princess Margaret Cancer Centre, Toronto, Ontario, Canada

^8^Laboratory Medicine Program, University Health Network, Toronto, Ontario, Canada

^9^Department of Laboratory Medicine and Pathobiology, University of Toronto, Toronto, Ontario, Canada.

^10^Department of Molecular Genetics, University of Toronto, Toronto, Ontario, Canada

^11^Hans Messner Allogeneic Blood and Marrow Transplant Unit, Princess Margaret Cancer Centre, Toronto, Ontario, Canada

1. **Supplementary Materials & Methods**
2. **Description of immunophenotypes**

The leukemic cells at diagnosis were comprised of two major subsets including blasts (24%) and monocytes (53%). Over 50% of the blasts at diagnosis, identified by homogenous CD13, CD33, and dim CD45 expression, and low side scatter were positive for CD11c, CD13, CD33, CD38, and CD117. There was a lower proportion of cells expressing CD7, CD11b, CD11c, CD64, CD123, HLA-DR, and the immature marker CD34 (2% of the cells). Of note, there was a subset of cells that co-expressed CD7 and CD34. The presence of *FLT3*-ITD was identified by two methods. In contrast to what had been observed at diagnosis, the proportion of bone marrow cells morphologically classified as blasts had markedly increased to 83% and showed a phenotypic shift with nearly 100% of the blasts in the relapse sample expressing CD34, CD117, CD123, and HLA-DR. In addition, the aberrant expression of CD7 changed from partial to all blast cells being positive; a small subset of the relapse blasts retained the expression of mature myeloid markers such as CD11b.

1. **Description of course of treatment**

The patient achieved complete remission after induction chemotherapy with daunorubicin and cytarabine, in combination with midostaurin. However, the patient relapsed 2 months after having completed 4 cycles of high dose cytarabine and midostaurin consolidation; the duration of CR was 6 months (Figure S1). Despite reinduction and salvage therapies, including gilteritinib, the patient died from the disease.

1. **Procedures of single-cell proteogenomic sequencing**

Cryopreserved cell suspensions were thawed according to Mission bio Tapestri Single-Cell DNA + Protein Sequencing User Guide QRC PN_6869G1 (Mission Bio, South San Francisco, CA, USA). 100ul of a sample at 8000 cells/µl was prepared in DPBS w/o Ca2+/Mg2+ (1X) (Gibco 14190-144) (Thermo Fisher Scientific, Waltham, MA, USA). Only samples with greater than 80% viability were selected. Cells were incubated with Human Tru Stain FcX (Biolegend, San Diego, CA, USA, Cat # 422301) for 3 minutes at RT for blocking and then stained with 10µl of antibody-oligo conjugate cocktail at RT for 30 minutes. Following washing, cells were suspended in a Cell buffer with a final sample concentration between 3000-4000 cells/µl. 35µl of the sample was used for encapsulation in the Tapestri instruments. Encapsulated cells were lysed, digested, and barcoded, followed by targeted PCR amplification in the presence of antibody tag primer, with PCR selected for 101-200 amplicon panel and purified. DNA bound to AMPure XP Reagent (Beckman Coulter, Brea, CA, USA, Product No. A63880) and antibody tags in the supernatant were further purified with Streptavidin Beads (Thermo Fisher Scientific, Waltham, MA, USA, Product No. 65001) and quantified using the Qubit dsDNA HS Assay Kit on the Qubit 2.0 Fluorometer (Invitrogen, Waltham, MA, USA) in the range of 0.2-4.0ng/µl.

Library preparation was carried out according to the Mission bio’s protocol and libraries were sized on Agilent Bioanalyzer High Sensitivity DNA kit chip at 450pb and 150pb respectively for DNA and protein Library. The library size was determined using Agilent High Sensitivity DNA Kit on the Bioanalyzer instrument (Agilent Technologies, Santa Clara, CA, USA) and quantified using the Qubit dsDNA HS Assay Kit on the Qubit 2.0 Fluorometer (Invitrogen, Waltham, MA, USA). 1.5 nm pool was denatured using 0.2N NaOH according to Illumina guidelines for 8 minutes at room temperature. The library pool was further diluted to 350 pM using 400mM Tris-HCl. This multiplexed pool was sequenced with the following parameters with an SP flow cell on the NovaSeq 6000 platform (Illumina, San Diego, CA, USA) standard run: Index Read 1: 1-8 cycles, Index Read 2: 1-8 cycles, Read 1: 1-151 cycles and Read 2: 1-151 cycles.

1. **Data filtering**

Raw sequencing reads were processed using Mission Bio’s Tapestri Pipeline (v2) and GQ.tsv, DP.csv, AF.csv, protein_reads.csv were exported from the Tapestri Insights. After filtering, 2,367 and 2,611 cells with high-confidence genotyping calls at all considered mutation loci were retained for downstream analyses in the diagnosis and relapse samples respectively (39.6% and 19.8%) (Table S3). To detect *FLT3*-ITD, FLT3_ITD_Ext was run for every single cell from both diagnosis and relapse samples as well as bulk DNA sequencing data(Tsai *et al*, 2020). For all mutations including *FLT3*-ITD, a gene was considered mutated only if the mutations were supported by at least 10% of the reads for that gene, in a single cell (≥ single-cell VAF (scVAF) 10%). Before arc-hyperbolic-sine transformation, protein abundance was normalized using ZINB-Wave and DeSeq2 (Risso *et al*, 2018; Love *et al*, 2014). After filtering out cells with low depth and genotype quality (genotype quality < 30 or sequencing depth < 10x) in any of the 3 mutated loci (chr5:170837545, chr11:32417910, and chr13:28592642) and with an average coverage of less than 15x or over 150x for the amplicon covering exon 14 of *FLT3* (AML_v2_FLT3_28608210 in the Mission Bio’s AML panel, covering chr13:28608210-28608371), 2367 and 2611 cells with high-confident genotype calls at all considered loci were retained for downstream analyses in diagnostic and relapse samples (40.9% and 20.7%). For all mutations including *FLT3*-ITD, only mutations supported by at least 10% mutant reads were considered mutated.

1. ***FLT3-ITD* detection and phylogenetic analyses**

For each single cell, mapped reads after filtering out reads with more than 3 mismatches and unmapped reads were combined into one bam file (Jun *et al*, 2015; Li *et al*, 2009). The combined bam file was processed using FLT3_ITD_Ext (with “-d -a 0 -t all -n amplicon -mr 2”)(Tsai *et al*, 2020). FLT3_ITD_Ext made 1343 ITD calls in 1325 cells and 12214 ITD calls in 12214 cells in diagnosis and relapse samples. Once *FLT3*-ITD was called for all cells, ITD calls supported by less than 10 reads or occurred only in one cell are filtered out. After filtering, 1239 ITD calls in 1222 cells and 4550 ITD calls in 4550 cells in diagnosis and relapse samples were retained for further analyses. We then performed multiple sequence alignments on all retained *FLT3* + ITD sequences (unique sequences) and the wildtype *FLT3* sequence using MAFFT (version 7.475) (Katoh & Standley, 2013). To categorize ITD and wildtype sequences using a phylogenetic tree, we clipped alignments from a position with the first gap to the last gap (+/- 5bp), resulting in 52bp-long alignment in this case. Distances between ITD sequences were computed using dist.dna function with “indel” model from the R package “ape” and the phylogenetic tree was generated using hclust (with “average” method) and ggtree (Yu *et al*, 2017; Paradis & Schliep, 2018). To match ITDs with their correct locations, ITDs in each branch (21bp and 39bp) were separately re-aligned with wild-type *FLT3* sequence. Sequence logos used in the Figure 1B, which accounts for the number of occurrences of each ITD were generated using WebLogo3. (Crooks *et al*, 2004)

1. **SNP array analysis**

High molecular weight DNA was extracted from the relapse sample and treated according to the manufacturer's instructions. Labeled DNA was hybridized to an Illumina CytoSNP-12 Beadchip (Illumina, San Diego, CA, USA) and scanned. Scanning was performed on an Illumina iScan instrument and data was imported into BlueFuse Multi Analysis software to analyze copy number and loss of heterozygosity.

1. **Analysis of protein expression**

To normalize protein expressions, ZINB-WaVE (with K=2, epsilon=1000, X=”~sample.source”, observationalWeights = TRUE, BPPARAM=DoparParam()) and DeSeq2 (with design =”~sample.source+clone” in DESeqDataSet function and sfType="poscounts", useT=TRUE, minmu=1e-6, and minReplicatesForReplace=Inf in DESeq function) were used(Risso *et al*, 2018; Love *et al*, 2014).

1. **Supplementary Figures**

**Figure S1.** Summary of clinical course, treatments, and molecular tests for the presented AML case. Abbreviations: 7/3= cytarabine (7 days) + daunorubicin (3 days); HDAC= high dose cytarabine; FLAG/Ida= fludarabine + cytarabine + G-CSF + idarubicin.

**Figure S2**. Comparison of variant allele frequencies between bulk DNA sequencing (x-axis) and single-cell proteogenomic sequencing (y-axis) found a very high correlation between allele frequencies measured by two approaches (Spearman’s Rho ~ 0.997, p-value < 5.16e-10).

**Figure S3.** Comparisons of single-cell variant allele frequencies for 5 considered mutations from (A) diagnosis and (B) relapse samples. As *FLT3*-D835Y and 39bp *FLT3*-ITD were not detected in the relapse sample, their single-cell variant allele frequencies were 0 and were omitted in the figure.

**Figure S4.** Single-cell variant allele frequencies for 1,942 cells from diagnosis sample according to their clone assignment. Among 1,942 cells, (A) 97 cells were assigned to C1 (*WT1*^mut^ and *NPM1*^mut^), (B) 1167 cells were assigned to C2 (*WT1*^mut^, *NPM1*^mut^, *FLT3*-TKD^mut^), (C) 287 cells were assigned to C3 (*WT1*^mut^, *NPM1*^mut^, 21bp *FLT3*-ITD), and (D) 111 cells were assigned to C4 (*WT1*^mut^, *NPM1*^mut^, 39bp *FLT3*-ITD). (E) The remaining 280 cells did not carry any of the 5 considered mutations and based on cell surface phenotype were predominantly T-cells.

**Figure S5.** Single-cell variant allele frequencies for 2,226 cells from the relapse sample according to their clone assignment. Among 2,226 cells, (A) 1,925 cells were assigned to C3R (*WT1*^mut^, *NPM1*^mut^, 21bp *FLT3*-ITD with CN-LOH at the *FLT3* locus) and (B) 301 cells that did not carry any of the 5 considered mutations.

**Figure S6.** Expression profiles of 16 cell surface proteins for 4,168 cells according to their clone assignments using heatmaps. Each row indicates each protein (CD117, CD123, CD34, CD7, CD38, CD33, CD11b, CD13, CD56, CD19, CD3, CD4, CD90, HLA-DR, CD45, and CD45RA) and each column represents a single cell. The color scheme indicates the expression level of each protein in each cell, where dark blue indicates no expression and dark red indicates high expression. Expression levels are asinh-transformed measures of normalized counts.

**Figure S7.** Expression profiles of 16 cell surface proteins in each clone (C1-4, C3R, and WT) using violin plots.

**Figure S8.** Distribution of homozygous 21bp *FLT3*-ITDs (single-cell variant allele frequencies over 95%) from diagnosis samples according to cell surface phenotype. Out of 98 cells (out of 287 cells) with immature myeloid cell-like signatures, 41 cells (41/98 cells, 42%) carried homozygous 21bp *FLT3*-ITD whereas only 11/189 cells (5.8%) carried homozygous 21bp *FLT3*-ITD among 189 cells with monocyte-like signatures. For this figure, cells assigned to other clones or from the relapse sample were masked.

**Figure S9.** Expression profiles of 16 cell surface proteins of C3 and C3R cells according to zygosity of 21bp *FLT3*-ITD. In each violin plot, expression levels of each cell surface protein for all C3 cells (287 cells), C3 cells with heterozygous (less than 95% scVAF) 21bp *FLT3*-ITD (98 cells), C3 cells with homozygous (greater than equal to 95% scVAF) 21bp *FLT3*-ITD (189 cells), and C3R cells (1,925 cells) are described.

1. **Supplementary Tables**

**Table S1.** List of 20 genes in the AML panel and 16 cell surface proteins targeted in this study

**Table S2.** Single cell proteogenomic sequencing metrics for two samples included in this study.

**Table S3.** Filtering procedures for 5,973 cells and 13,215 cells from diagnosis and relapse samples and remaining cells after each filtering step

**Table S4.** Clinical characteristics of the AML patient included in this study at diagnosis and relapse.

**Table S5.** Summary of 5 mutations considered in this study and their allele frequencies and mutated cell fraction using bulk cell sequencing and single-cell sequencing.

**References**

Crooks, G.E., Hon, G., Chandonia, J.-M. & Brenner, S.E. (2004) WebLogo: A Sequence Logo Generator. *Genome Research*, 14, 1188–1190.

Jun, G., Wing, M.K., Abecasis, G.R. & Kang, H.M. (2015) An efficient and scalable analysis framework for variant extraction and refinement from population-scale DNA sequence data. *Genome Research*, 25, 918–925.

Katoh, K. & Standley, D.M. (2013) MAFFT Multiple Sequence Alignment Software Version 7: Improvements in Performance and Usability. *Molecular Biology and Evolution*, 30, 772–780.

Li, H., Handsaker, B., Wysoker, A., Fennell, T., Ruan, J., Homer, N., Marth, G., Abecasis, G. & Durbin, R. (2009) The Sequence Alignment/Map format and SAMtools. *Bioinformatics*, 25, 2078–2079.

Love, M.I., Huber, W. & Anders, S. (2014) Moderated estimation of fold change and dispersion for RNA-seq data with DESeq2. *Genome biology*, 15, 31.

Paradis, E. & Schliep, K. (2018) ape 5.0: an environment for modern phylogenetics and evolutionary analyses in R. *Bioinformatics*, 35, 526–528.

Risso, D., Perraudeau, F., Gribkova, S., Dudoit, S. & Vert, J.-P. (2018) A general and flexible method for signal extraction from single-cell RNA-seq data. *Nature Communications*, 9, 284.

Tsai, H.K., Brackett, D.G., Szeto, D., Frazier, R., MacLeay, A., Davineni, P., Manning, D.K., Garcia, E., Lindeman, N.I., Le, L.P., Lennerz, J.K., Gibson, C.J., Lindsley, R.C., Kim, A.S. & Nardi, V. (2020) Targeted Informatics for Optimal Detection, Characterization, and Quantification of FLT3 Internal Tandem Duplications Across Multiple Next-Generation Sequencing Platforms. *The Journal of Molecular Diagnostics*, 22, 1162–1178.

Yu, G., Smith, D.K., Zhu, H., Guan, Y. & Lam, T.T. (2017) ggtree: an r package for visualization and annotation of phylogenetic trees with their covariates and other associated data. *Methods in Ecology and Evolution*, 8, 28–36.
