# Supplementary material for: Single cell proteogenomic sequencing identifies a relapse‐fated AML subclone carrying FLT3‐ITD with CN‐LOH at chr13q
Source: EJHaem. 2022 Feb 24;3(2):426–33. doi: 10.1002/jha2.390 (PMC9175792; doi:10.1002/jha2.390)
Supplement: Supplementary file 14 — Table S4 [file JHA2-3-426-s003.docx]

**Table S4. Summary of clinical information**

|  | | **Diagnosis** | **Relapse** |
| --- | --- | --- | --- |
| PB/BM findings | WBC (x10^9^/L)  Hb (g/dL)  Platelet (x10^9^/L)  PB blast (%) | 106  7.3  75  28 | 51.1  7.1  48  39 |
|  | BM cellularity | Hypocellular | Hypercellular |
|  | BM blast (%) | 41 | 83 |
| Molecular tests | *FLT3*-ITD^a^ | Low positive ^a^  (Multiple peaks, 21bp with ITD level of 13% and 39bp with ITD level of 3.5%.) | High positive ^a^  (Single peak, size: 21bp with ITD level of 91.37%) |
|  | *FLT3*-TKD^a^ | Detected | Undetectable |
|  | *NPM1*^a^ | Detected | Detected |
|  | NGS | *WT1*, S386* (VAF 43.4%)  *NPM1*, W288Cfs*12 (VAF 1.4%)  *FLT3*, D835Y (VAF 34.7%)  21bp *FLT3*-ITD (VAF 8.8%)  39bp *FLT3*-ITD (VAF 2.8%) | *WT1*, S386* (VAF 45.0%)  *NPM1*, W288Cfs*12 (VAF 36.0%)  21bp *FLT3*-ITD (VAF 88.4%)  *FLT3* D835Y and 39bp *FLT3*-ITD were not detected. |
| Cytogenetics | G-banding | 46, XX [24] | Not done |

PB, peripheral blood; BM, bone marrow; CBC, complete blood cell count

^a^ Tested by PCR-PFLP. Mutation percentage was calculated.
